# Peanut allergen Ara h 6 is detectable in blood transfusion products

**DOI:** 10.1002/clt2.12307

**Published:** 2023-11-03

**Authors:** Fleur A. C. Jansen, Klaske van Norren, Joseph L. Baumert, Annegeet van den Bos, Joannes F. M. Jacobs, Stef J. Koppelman

**Affiliations:** ^1^ Wageningen University & Research Wageningen The Netherlands; ^2^ University of Nebraska‐ Lincoln Lincoln Nebraska USA; ^3^ Department of Laboratory Medicine Radboud University Medical Center Nijmegen The Netherlands

To the Editor,

Peanut allergen Ara h 6 is known to maintain IgE‐binding capacity upon exposure to digestive enzymes^1^ and its presence in circulation after consumption of peanut has been demonstrated.^2^, ^3^ Therefore, it has been speculated that food‐derived allergens could be transferred via blood transfusion products, causing an allergic reaction in food‐allergic recipients.^4^, ^5^ However, in published case reports, presence of food allergen in donated material could not be confirmed due to lack of remaining transfusion material and/or lack of sensitive analytical methods. Using a newly developed sensitive immune‐assay for detecting Ara h 6 in human serum, we now report to what extent consumed peanut allergens can be present in blood transfusion materials and estimate the associated risk for peanut‐allergic recipients.

When five donors consumed peanut prior to blood donation (all donors gave informed consent to use their blood samples for clinical research, intervals ranging from 4 to 16 h; see methods in Supplementary Information), serum Ara h 6 levels were elevated up to 14 h after consumption (Figure 1). The highest serum Ara h 6 level was 4.20 ng/mL (±1.43 SEM), measured in serum collected 5 h after consumption.

**FIGURE 1:**
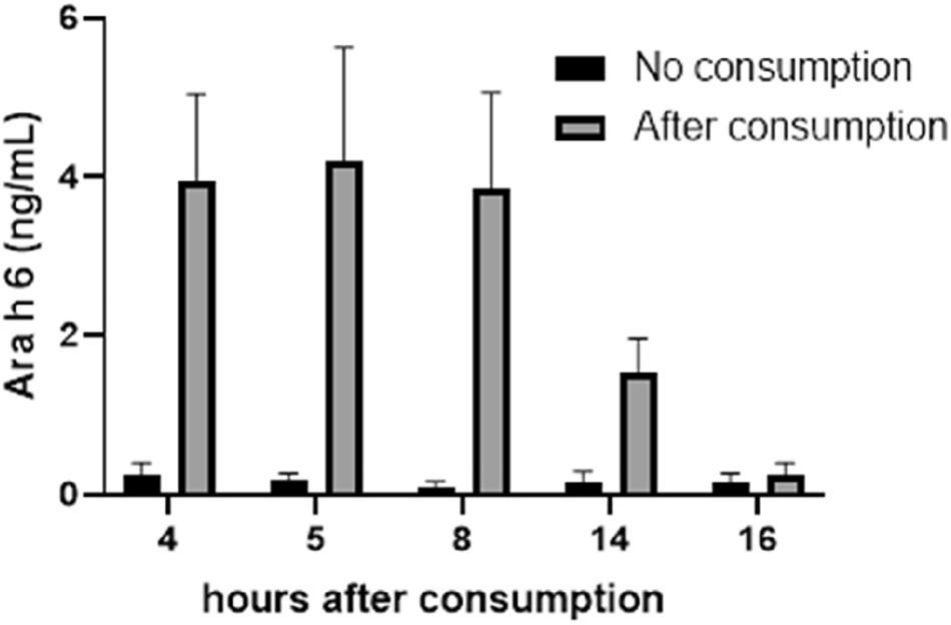
Ara h 6 serum levels (ng/mL) after being restricted from eating any peanut containing foods for 48 h (No consumption) and after consuming 200 g of peanut (After consumption). Time between peanut consumption and serum collection differed between the five individuals and ranges from 4 to 16 h. Unpaired multiple comparison *t*‐test was performed and showed that only the Ara h 6 concentration 16 h after consumption did not differ from baseline (*p* = 0.675). Samples were analyzed by three independent ELISAs and data is presented as mean ± SEM.

We use an immunoassay (sandwich ELISA) to detect Ara h 6, and while this demonstrates that Ara h 6 in circulation possesses IgG epitopes, this does not necessarily mean that Ara h 6 in circulation still has allergenic activity. Such can be shown by basophil activation tests, however, within the current setting at our laboratories there was no access to those tests. Others have shown that levels of Ara h 6 detected in circulation after peanut consumption correlate with basophil activation potency^3^ showing that Ara h 6 in circulation is still allergenic, but for our current study we do not have this proof, which is a limitation of our study.

Adhering to clinical guidelines for routine blood donation, 320 mL units of plasma were also obtained from these donors, at the same intervals after peanut consumption, and a similar pattern of Ara h 6 appearance was observed (Supporting Figures S1 and S2).

These data were obtained from donors that consumed at one eating occasion a relatively large amount of peanut. To get more insight into the clinical relevance of this observation, plasma samples from 20 adult subjects and a plasma omni pool product obtained from 600 donors (which is a routine transfusion product for clinical use) were analyzed for Ara h 6 content. As per blood donation guidelines, donors had no dietary restrictions and did not receive instruction to consume or avoid peanut. In 17 of the 20 individual plasma samples, Ara h 6 was detected (Figure 2). In eight of these samples, values above LLOQ were measured and two of these samples showed fairly high Ara h 6 levels: 1.09 ng/mL (±0.043 SEM) and 0.66 ng/mL (±0.066 SEM). Differences in Ara h 6 levels across these donors can be due to amount of peanut ingested or due the time between peanut ingestion and blood donation (or a combination of both factors).

**FIGURE 2:**
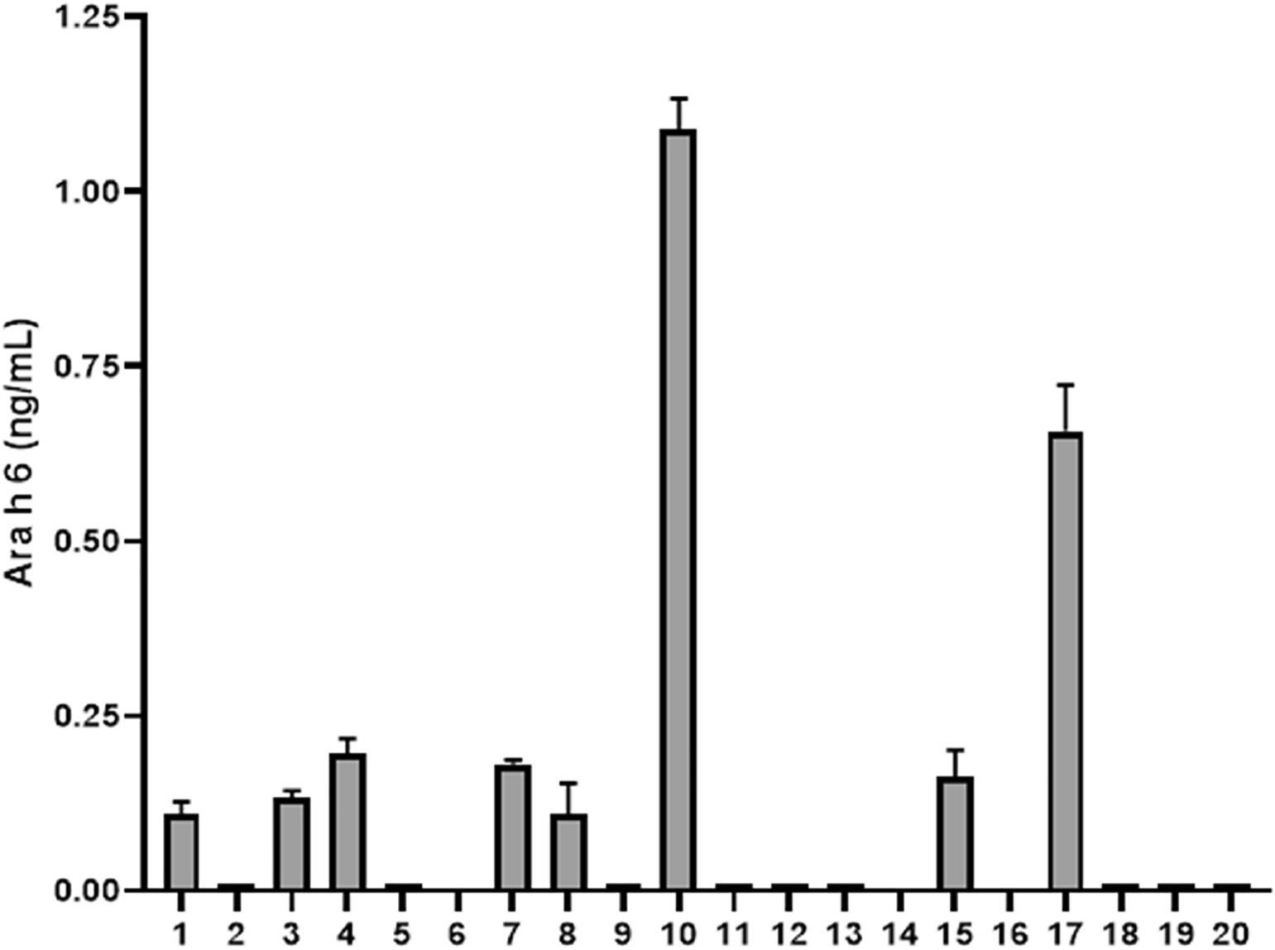
Ara h 6 levels (ng/mL) in plasma samples donated for blood transfusion. Of the 20 plasma samples analyzed, eight samples showed values above LLOQ. These values were obtained by three independent ELISAs and depicted as mean ± SEM.

In the plasma omni pool product, 0.56 ng/mL (±0.15 SEM) of Ara h 6 was found.

It is not known which concentration of peanut allergen in circulation is needed to trigger a systemic reaction in vivo, and if such levels can be reached by blood transfusion, but an estimation can be made using thresholds for basophil activation reported in literature. For example, the in vitro study of Hemmings et al.^6^ showed that 0.1–0.18 ng/mL Ara h 6 triggered basophil degranulation and an ex vivo study of Mose et al.^3^ showed that human serum samples obtained after consumption of peanut, containing 0.025–0.05 ng/mL Ara h 6, can degranulate passively sensitized basophils. However, reported threshold values differ, probably due to differences in methodology, but also due to inter‐patient variability of the sensitivity of mast cells and basophils to Ara h 6. The plasma omni pool product investigated in this study would be used in clinical practice at 12–15 mL per kg bodyweight (according to its product data sheet), resulting in a dilution of approximately 3.5–4.5‐fold in a recipient. This would result in a modeled Ara h 6 concentration of 0.12–0.16 ng/mL in the recipient's circulation.

These modeled concentrations of Ara h 6 are in the same order of magnitude as, or somewhat above, the threshold for basophil activation, suggesting that transfusion using a pooled plasma product can trigger an allergic reaction in peanut‐allergic recipients. At this stage, no clinical data are available to demonstrate reactivity of Ara h 6 in transfusion products. For example, the role of donor and recipient IgG, which may prevent Ara h 6 to bind to effector cells, needs further exploration. Follow‐up studies could include basophil activation assays with (Ara h 6‐positive) transfusion material, or similar in vivo experiments with peanut‐sensitized animals.

The importance of other digestion‐resistant peanut allergens, like Ara h 2, should be explored as well, as these allergens can further potentiate the allergenic load of transfusion products. For now, only Ara h 6 was considered because no methods are available to detect and quantify other peanut allergens in blood samples.

## AUTHOR CONTRIBUTIONS

**Fleur A. C. Jansen**: Data curation (lead); investigation (lead); validation (equal); visualization (equal); writing—original draft (equal). **Klaske van Norren**: Methodology (equal); supervision (equal); writing—review and editing (equal). **Joseph L. Baumert**: Conceptualization (equal); writing—review and editing (equal). **Annegeet van den Bos**: Resources (equal); writing—review and editing (equal). **Joannes F. M. Jacobs**: Resources (equal); writing—review and editing (equal). **Stef J. Koppelman**: Conceptualization (equal); methodology (equal); supervision (equal); validation (equal); writing—original draft (lead).

## CONFLICT OF INTEREST STATEMENT

Fleur A. C. Jansen, Klaske van Norren, Joseph L. Baumert, Annegeet van den Bos and Joannes F. M. Jacobs report no conflict of interest. Stef J. Koppelman is consultant to DBV Technologies, outside the scope of the current paper.

## Supporting information

Supporting Information S1

## Data Availability

Research data are not shared.
