# Supplementary material for: Peanut allergen Ara h 6 is detectable in blood transfusion products
Source: Clin Transl Allergy. 2023 Nov 3;13(11):e12307. doi: 10.1002/clt2.12307 (PMC10624233; doi:10.1002/clt2.12307)
Supplement: Supplementary file 1 — Supporting Information S1 [file CLT2-13-e12307-s001.docx]

**Supporting Information: Methods**

*Clinical Pilot Study*
Serum and plasma were obtained from five donors who consumed peanut, adhering to the general procedures of the blood bank (Sanquin, Nijmegen, The Netherlands) concerning donation of blood for transfusion purposes. All donors gave informed consent to use their blood samples for clinical research. From each donor, blood was collected twice: after being restricted from any peanut-containing food for 48 hours and after consuming 200 g of light-roasted, unsalted peanuts obtained from a local supermarket (Albert Heijn; private label product). The time between peanut consumption and blood collection was different for each subject and varied between 4 and 16 hours. Therefore, although different time intervals after peanut consumptions were used, it should be noted that this does not represent a true time course, because the samples at different time points were obtained from different donors. This was because a full-size plasma unit (i.e. 320 ml) was collected at a set timepoint, to comply with routine blood donation procedures, preventing us from taking multiple samples per donor. After production of plasma and serum, following the guidelines of Good Clinical Practice, materials were transferred back from the blood bank to the clinical research unit. Materials were used in a de-identified form, in accordance with local guidelines and regulations and complying with the Declaration of Helsinki and the Good Clinical Practice guidelines. Both plasma and serum samples were aliquoted and stored at -80°C until further analysis.

*Clinical samples*
Plasma samples from twenty adult subjects and a plasma omni pool product obtained from 600 donors, who had no dietary restrictions or instructions to consume or avoid peanut, were obtained from Sanquin Blood Bank. All subjects gave informed consent to donate their blood samples for research, and samples were used in a de-identified form, in accordance with local guidelines and regulations and complies with the Declaration of Helsinki and the Good Clinical Practice guidelines. The samples were aliquoted and stored at -80°C until further analysis.

*Ara h 6 ELISA*
The quantification of Ara h 6 in the serum and plasma samples was performed with a sandwich ELISA using the Ara h 6 monoclonal antibody 3B8 and biotinylated monoclonal antibody 3E12 of Indoor Biotechnologies (Cardiff, UK). The monoclonal antibody 3B8 was diluted in 50mM carbonate-bicarbonate buffer, pH 9.6 (1:1000) and used to coat Nunc Maxisorp flat-bottom 96-well plates (Invitrogen, Breda, NL), 100 µL/well. After overnight incubation at 4°C, wells were washed three times with 300 µL PBS (pH 7.4; 136.89 mM NaCl, 1.47 mM KH_2_PO_4_, 8.10 mM Na_2_HPO_4_, 2.68 mM KCl) containing 0.05% Tween 20 (PBS-T) and then blocked for 30 min with PBS containing 1% (w/w) bovine serum albumin (BSA; A7030; Sigma-Aldrich, Zwijdrecht, NL), 200 µL/well. Plates were washed again, whereafter standards, blanks, and samples were added in duplicate to the plate, 100 µL/well. Before adding them to the plate, based on our previous research^1^, samples were pretreated to dissociate the potentially present IgG from Ara h 6 by diluting the samples four-times in 1% BSA-PBS and heating them for 20 min at 75 °C. To obtain the standards, naturally processed Ara h 6, which reportedly better represents Ara h 6 after gastrointestinal digestion^2^, was 3-fold serial-diluted in 1% BSA-PBS from 10 to 0.014 ng/mL. After one hour incubation at room temperature, plates were washed with PBS-T and the biotinylated monoclonal antibody 3E12 diluted in 1% BSA-PBS (1:1000) was added, 100 µL/well. Next, Streptavidin-HRP diluted in 1% BSA-PBS (1:5000) was added, 100 µL/well, after 1 hour incubation at room temperature and washing with PBS-T. The plate was incubated with Streptavidin-Peroxidase for 1 hour at room temperature and washed before adding TMB, supersensitive (T4444; Sigma-Aldrich, Zwijndrecht, NL), 100 µL/well, to stain the plate. This substrate was incubated for 10 minutes at room temperature in the dark before the staining was stopped by adding 1 M H_2_SO_4_, 50 µL/well. Absorbance was measured at 450 nm by SpectraMax M2 (Molecular Devices, San Jose, CA, USA). All samples were measured by three independent ELISAs.

*Data analysis*

GraphPad Prism 9 (San Diego, CA, USA) was used to interpolate the data and design the graphs. Interpolation from the standard curve was performed with a curve fit based on the asymmetric sigmoidal model, X is concentration. All data is shown as mean ± SEM. Unpaired multiple comparison t-test was performed to test for significant differences between Ara h 6 serum concentration before and after peanut consumption at the different time intervals. P-value below 0.05 was considered statistically significant. The Lower Level of Detection (LLOD) and Lower Level of Quantification of Ara h 6 in the samples were determined to be 0.024 and 0.10 ng/mL, respectively. These values were defined as the interpolated value corresponding to OD(5 SD_blanks_) and OD(10 SD_blanks_), corrected for dilution factor.

**Supporting Information: Results**

Previously^1^, the detection method used in this paper was only applied to serum. To check whether this method can also be used for detection in plasma, a spike- and recovery test was performed. Plasma, collected after the donor being restricted from consuming any peanut containing foods for 48hr, was spiked with Ara h 6 standard at concentrations of 10 – 1.1 – 0.12 - 0.014 ng/mL. Subsequently, the ELISA protocol for serum was followed to quantify the Ara h 6 levels in these spiked samples. Supporting Figure 1 shows the standard curve used to interpolate the obtained OD values (left) and the Ara h 6 recovery graph (right). On average, the detected Ara h 6 concentrations were overestimated by 25%, but the duplicate measurements show that, except for the lowest concentration spiked, the quantification can be considered precise (R^2^=0.999). No interference by plasma components was observed, allowing the method to be used for analyzing plasma samples.


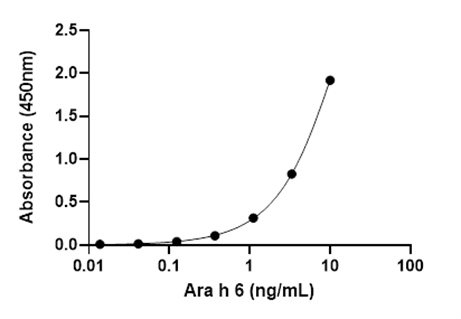

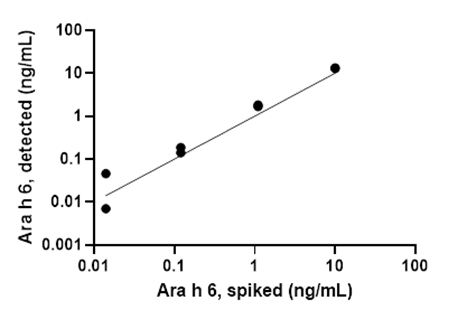


**Supporting Figure 1 Sandwich ELISA standard curve (left) and recovery graph of spiked plasma samples (R^2^=0.999) (right).** Left Panel: standard curve in buffer. Right Panel: Plasma, collected after the donor being restricted from consuming any peanut containing foods for 48 hours, was spiked with Ara h 6 (10 – 1.1 – 0.12 – 0.014 ng/mL) and measured in duplicate by sandwich ELISA. Values were interpolated with a curve fit based on the asymmetric sigmoidal model, X is concentration. Duplicate values are plotted against the spiked concentration.

In Supporting Figure 2, Ara h 6 levels in plasma samples quantified by the current ELISA method are shown. When plasma samples were analyzed for Ara h 6 by the ELISA method, a similar pattern was observed as for the serum samples previously shown in Figure 1 (main text). The values observed for the plasma samples are somewhat higher than for the serum samples, which is in line with the results of the plasma spike- and recovery test. The pattern that Ara h is increased up to 14 hours, reaching a plateau at 4-8 hours, and diminishing at 16 hours suggests that Ara h 6 levels start to return back to baseline from 8 hours after consumption. However, this interpretation should be made with caution as no data is available on the change of Ara h 6 levels over time within the individuals since a full-size plasma unit (i.e. 320 ml) was collected at a set timepoint to comply with routine blood donation procedures.


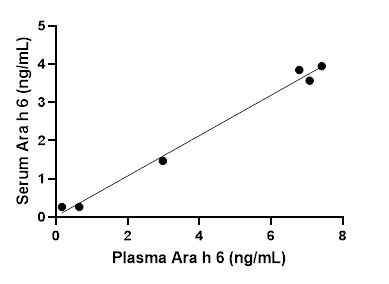

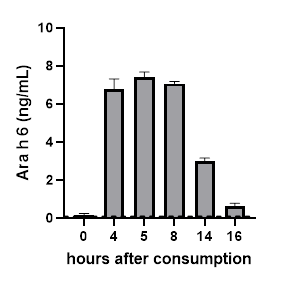


**Supporting Figure 2 Ara h 6 plasma levels (ng/mL) after consuming 200 g of peanut (left) and correlation plot of average Ara h 6 levels measured in plasma and serum** **(R^2^=0.991) (right).** Time between peanut consumption and plasma collection differed between the individuals, ranging from 4 to 16 hours. Ara h 6 level depicted as 0 hours after peanut consumption corresponds to plasma drawn after a donor being restricted from peanut consumption for 48 hours. In this case, this sample was obtained from the same donor as the sample collected 4 hours after consumption. Plasma samples were analyzed by three independent ELISAs and data is presented as mean ± SEM.

**Supporting Information: Additional References**

1. Koppelman SJ, Witteveen M, JanssenDuijghuijsen L, Baumert JL, Witkamp RF, van Norren K. Detection of peanut allergens in serum: circumventing the inhibitory effect of immunoglobulins. *Allergy*. 2020;75(7):1835-1836. doi:10.1111/ALL.14330

2. Apostolovic D, Stanic-Vucinic D, de Jongh HHJ, et al. Conformational stability of digestion-resistant peptides of peanut conglutins reveals the molecular basis of their allergenicity. *Sci Rep*. 2016;6. doi:10.1038/SREP29249
